# NK cells in asthma exacerbation

**DOI:** 10.18632/oncotarget.4841

**Published:** 2015-07-08

**Authors:** Lars Lunding, Michael Wegmann

**Affiliations:** Division of Mouse Models of Asthma, Priority Area Asthma & Allergy, Research Center Borstel (RCB), Borstel, Germany

**Keywords:** asthma, interleukin 17, exacerbation, NK cells, virus, immunology and microbiology section, immune response, immunity

Asthma management typically aims at maintaining asthma control by using corticosteroids that suppress airway inflammation. However, acute asthma exacerbations often occur leading to loss of control and necessitating systemic corticosteroids, hospitalization, or even both. Consequently, acute asthma exacerbations have a major impact on the mortality and morbidity of asthma as well as on the medical costs associated with the disease [1]. Although the development of new medications to reduce the frequency and severity of acute asthma exacerbations has advanced recently, an incomplete understanding of the immuno-pathogenetic mechanisms underlying these episodes of spontaneous worsening still limits their development.

Epidemiological surveys suggested respiratory viral infections as the most common triggers of asthma exacerbation [2]. In most cases these viral infections are caused by rhino viruses and respiratory syncytial virus, while infections with influenza virus, human metapneumovirus, corona virus, or parainfluenza viruses have been recorded less frequently [2]. All these viruses have in common that their genome is encoded on single-stranded (ss) RNA, which in turn is recognized by the vertebrate immune system via toll-like receptor 3 (TLR3) and retinoic acid induced gene I (RIG-I).

We therefore hypothesized that stimulation of TLR3/RIG-I could be a critical event in the pathogenesis of acute, virus-induced asthma exacerbations. In order to test this hypothesis we delivered the synthetic TLR3/RIG-I ligand poly IC to the lungs of mice sensitized to ovalbumin (OVA) after experimental allergic asthma has been established by inhalation of an OVA-aerosol. Indeed, this “second hit” resulted in acute worsening of the disease by points of mucus production, lung function, and airway inflammation. Interestingly, the inflammatory infiltrate was not only characterized by significantly enhanced numbers of eosinophils but also by a pronounced influx of neutrophils, which have been attributed to corticosteroid-resistant asthma. The aggravated inflammatory response was further associated with elevated production of various cytokines including typical T helper 2 (TH2) type cytokines and TH17 type cytokines such as interleukin (IL) 17A. Using IL-17A deficient animals we could demonstrate that this cytokine is essential for the induction of poly IC-triggered exacerbation of experimental asthma in mice and subsequently we went on to identify the cellular source of IL-17A. After in-vitro restimulation we found increased numbers of TH17 cells in the lungs of mice with experimental asthma exacerbation. Adoptive transfer experiments in mice demonstrated that proinflammatory TH17 cells are capable of aggravating already established experimental asthma towards a corticosteroid-resistant phenotype by triggering neutrophil infiltration into the airways. However, since our protocol for the induction of an experimental asthma exacerbation also successfully worked in IL-23p19 deficient animals, which do not display mature, proinflammatory TH17 cells, we doubted the critical importance of TH17 in our model [3]. We therefore used homozygous *Rorc-*GFP mice that do not express functional RORγt and therefore are incapable to produce TH17 cells [4]. Again local application of poly IC aggravated allergic airway inflammation and induced airway neutrophilia in these mice, so that TH17 can definitely be excluded to be the pathologically relevant source of IL-17A in our model.

FACS analysis also revealed markedly enhanced numbers of IL-17A-producing natural killer (NK) cells, but not NK T cells, in the lungs of animals with experimental asthma exacerbation. Consequently, we depleted NK cells in these mice, which resulted not only in a reduction of IL-17A levels in broncho-alveolar lavage (BAL) fluid to the levels of the controls, but also ablated most hallmarks of acute asthma exacerbation [3]. These data suggest that NK cells drive the inflammatory response underlying acute asthma exacerbation by producing IL-17A. Thus, these data implement NK cells for the first time into the pathogenesis of virus-induced asthma exacerbations.

NK cells are more frequent in the lung than in other organs [5] and their role in protecting against respiratory infections by viruses has been broadly described in animal models. Also they can directly react on TLR3 activation by ssRNA without support of antigen-presenting cells [6]. However, whether these important and beneficial functions may lead to aggravation of an already existing asthma remains unclear. Alike T cells NK cells can be divided into different subsets such as NK1/NK17 cells, NK2 cells, or NKreg cells according to their cytokine production [7]. Whether NK1/NK17 cells that are characterized by producing interferon (IFN) γ and IL-17A are actually the source of IL-17A and regulate the inflammatory response resulting in asthma exacerbation in our model remains to be investigated. Since we did neither observe enhanced production of IFNγ in wild type animals with acute asthma exacerbation nor an effect of NK cell depletion on IFNγ levels in BAL fluid, this hypothesis remains doubtful. Our data further suggest that the NK cells in our model require rather IL-6 then IL-23 or RORγt to express IL-17A and to exert their pathological function. Of course ongoing studies that characterize this NK cell subset in points of cytokine production, chemotaxis, survival, and interaction with other immune cells are necessary, but will help understanding the pathologic functions of NK cells and their potential as therapeutic targets in virus-induced asthma exacerbations.

## References

[1] Ivanova JI (2012). J Allergy Clin Immunol.

[2] Jackson DJ (2011). J Allergy Clin Immunol.

[3] Lunding LP (2015). J Immunol.

[4] Lochner M (2008). J Exp Med.

[5] Grégoire C (2007). Immunol Rev.

[6] Schmidt KN (2004). J Immunol.

[7] Deguine J (2013). Immunol Rev.

